# Colectomy Followed by J-Pouch Reconstruction to Correct Total Colonic Aganglionosis

**DOI:** 10.3390/children9010101

**Published:** 2022-01-12

**Authors:** Konrad Reinshagen, Gunter Burmester, Johanna Hagens, Thomas Franz Krebs, Christian Tomuschat

**Affiliations:** 1 Department of Pediatric Surgery, University Medical Center Hamburg-Eppendorf, 20246 Hamburg, Germany; j.hagens@uke.de (J.H.); c.tomuschat@uke.de (C.T.); 2 Department of Pediatrics, Altonaer Kinderkrankenhaus, 22763 Hamburg, Germany; gunter.burmester@gmail.com; 3 Department of Pediatric Surgery, Children’s Hospital of Eastern Switzerland, 9006 St. Gallen, Switzerland; thomasfranz.krebs@kispisg.ch

**Keywords:** hirschsprung disease, Total Colonic Aganglionosis, J-pouch reconstruction, restora-tive proctocolectomy

## Abstract

Background: Patients suffering from complete colonic aganglionosis (TCA) require the best surgical care possible. Only a few studies reported J-Pouch repair as the primary reconstructive surgery in TCA patients. This study adds to the current literature a thorough clinical and functional outcomes group. Methods: Between 2011 and 2021, medical records of Hirschsprung disease (HD) patients who underwent J-Pouch reconstruction during infancy (n = 12) were reviewed. In close follow-up, bowel function and satisfaction with operation results were evaluated. The median age at the time of J-Pouch reconstruction was 16 months, and covering ileostomies were closed four months later. There were no postoperative problems. After the final repair, Pouch-related problems (PRP) occurred in 27% of the children and were treated conservatively. There was no histological evidence of pouchitis in any of the individuals. The median 24-h stooling frequency was 4–5 at the latest follow-up 51 months following enterostomy closure. Conclusions: The current study’s findings support the existing literature and advocate for J-pouch repair in TCA patients. However, more research will be needed to determine the best time to undergo pouch surgery and ileostomy closure in TCA patients.

## 1. Introduction

Complete colectomy is the only curative treatment for total colonic aganglionosis (TCA). However, the goal of such intensive surgical treatment must be followed by a reconstruction that leads in the long term to complete continence. Ravitch originally described colectomy with continent repair in adult patients in 1951 utilising a straight ileoanal anastomosis [1]. Unfortunately, the acquired continence was relatively limited, resulting in poor results. Sir Alan Parks and Nichols created the S-Pouch in 1978, allowing continent reconstruction following a total colectomy [2]. However, because the S-Pouch was difficult to reconstruct, it was not frequently employed [3]. Two years later, Utsonomiya popularised the J-Pouch reconstruction, which revolutionised pouch surgery primarily because it was easier to create while providing the obvious benefits of a pouch reservoir following colectomy [4]. Furthermore, the function outperformed the S-Pouch [3]. The objective of pouch surgery is not only to create a reservoir as a neorectum, but also to diminish prokinetic activity in the direction of the anal canal, resulting in increased continence and a decreased urge to defecate [5]. Systematic investigations of the quality of life in adult patients with over 4790 patients have revealed that the quality of life 12 months after reconstruction did not differ from the general population’s quality of life [6]. In adult medicine, pouch repair is the standard surgery for individuals who have had a total colectomy for a variety of reasons [3].

Publications in paediatric and adolescent surgery suggest that in patients with TCA, a straight ileoanal anastomosis is preferable to forming a pouch [7]. The key argument is that the small bowel adjusts to the increased need for fluid absorption and that the number of faeces per 24 h drops dramatically over a year. Another point is that pouch surgery causes much more problems, such as pouchitis [8,9]. On the other hand, recent evaluations show that pouch reconstruction procedures have the same complications and postoperative course [10]. This paper describes our data and methodologies for ileoanal pouch reconstruction in TCA patients.

## 2. Materials and Methods

The study followed the principles of the Helsinki Declaration and was approved by the local institutional review board Hamburg ethics committee (2021-300128-WF). The study’s goal was to characterise the outcomes of complete colectomy followed by primary J-pouch formation during childhood. From January 2011 to December 2021, all TCA children were screened in hospital records from the paediatric surgery departments at the University Center Hamburg-Eppendorf and Altona Children’s Hospital Hamburg.

The medical patient records were analysed to look at the presentation, diagnostic studies, operative approach (pouch length, laparoscopic assisted), complications, functional results, growth, and electrolytes status. Regular outpatient clinic visits were used to collect follow-up data. There were also questions about weaning age, urine continence, stool frequency, abdominal distension, nutritional status, and satisfaction with functional and cosmetic results. All data from eligible patients was recorded into a standardised spreadsheet. The study included all TCA patients who had undergone reconstructive surgery with a total colectomy and ileoanal J-pouch reconstruction. Patients who had J-Pouch reconstruction for reasons other than TCA and all patients with rectosigmoid HD were excluded. The primary goal was to evaluate faecal incontinence and pouchitis and enteritis-like symptoms. Presenting symptoms, perioperative complications and postoperative outcome were evaluated as binary variables (1 = yes, 0 = no). Patient demographics were calculated as median with maximum and minimum values given as total range. Statistical analysis was performed descriptively using Microsoft Office Excel (version 16.56, Microsoft, Seattle, WA, USA).

### Surgical Technique

Protective enterostomies were performed on all patients involved in this study (mean age six months). Total colectomy was performed via laparoscopy or laparotomy, followed by a transendorectal pull-through technique, depending on the number of previous procedures and associated intra-abdominal adhesions.

The colon was surgically prepared with caution to protect the ileocolic artery, which is critical in supplying the ileoanal J-pouch. The pouch reconstruction was done extracorporeally. The pouch length was determined using the criteria for visceral surgery; however, the required lengths were lowered to ensure healthy growth of the residual small intestine. As a result, we evaluated a pouch length of 5 cm to a maximum of 7 cm in infancy. Because of its smaller branches than a typical GIA-stapler, the J-pouch was created with the help of the EndoGIA.

The final rectal resection and ileoanal J-pouch anastomosis were performed transanally, with the dentate line and transition zone preserved using an anal retractor. TCA does not require a complete mucosectomy, which is needed for familial adenomatous polyposis (FAP) or ulcerative colitis (UC), lowering the risk of postoperative sensory problems. A Foley catheter was inserted, and was kept for several days after surgery since nerve irritation of the hypogastric plexus could cause temporary bladder palsy.

For four weeks, all patients were followed up weekly. Particular emphasis was paid to assessing the progression of anastomotic stricture, which may necessitate the use of bougienage at an early stage if necessary.

## 3. Results

### 3.1. Patient Database

From 2011 to 2021, nine boys and three girls received subtotal or total colectomy for long-segment HD followed by J-pouch repair (Table 1). Given that HD has a genetic foundation, it’s not surprising that two children in our research population reported familial clustering of HD [11]. Furthermore, one HD patient was diagnosed with Down Syndrome, and another with Herlyn-Mayer-Wunderlichs syndrome. Unilateral renal agenesis was described in one boy. One girl was also diagnosed with congential myopathy and dysplastic kidney.

Nine children (81%) showed typical symptoms during their neonatal period. Four children presented with constipation and five with failure to thrive throughout their first year of life, and they included not passing meconium, a distended abdomen, and bilious vomiting (Table 2). TCA was diagnosed in five children within the first month of life, four within the first year, one at the age of three, and one at the age of five. At the age of 15 months, a rectal mucosal biopsy failed to establish a diagnosis in one of those children. TCA was diagnosed after a biopsy revision in the fourth year of life. The other patient suffered from with persistent constipation and abdominal distension following surgery at another hospital that resulted in removing a 7 cm hypoganglionic segment. TCA was eventually revealed via biopsies at our clinic.

### 3.2. Age at Correction

Reconstruction was performed on patients ranging in age from 3 months to 5 years, with a mean of 16 months, and all patients underwent a temporary double-lumen ileostomy. Stoma closure was performed at an average age of 4 months (1, 5–11 months) after primary repair in the current cohort. There were periodic follow-up assessments following the primary repair, particularly for stool frequency and continence, urinary continence, nutrition, failure to thrive, and vitamin, electrolyte, and iron status. However, two of the patients are currently in school, while the rest are still in infancy.

### 3.3. Height and Weight

All of the patients in our sample were over the third percentile in terms of body weight and size (Table 2). Patients identified with HD after the newborn period, on the other hand, showed signs of failure to thrive. We noted that all patients crossed their percentiles downwards in height and weight before repair. Postoperative catch-up growth was accomplished in the majority of the children. At four years of age, only one out of eleven patients is dystrophic and shows scores in the second percentile for height and weight.

### 3.4. Stool Frequency & Continence

After closure of the protective stoma, we observed a stool frequency of 15–20 bowel movements per day, resulting in electrolyte and fluid loss as well as skin irritation in the perianal region. To manage bowel movements, some patients required loperamide medication. Overall, all patients had decreased stool rates of roughly 4–5 bowel movements per day throughout time. Today, six of nine patients above the age of 3 years are entirely continent, and two of 9 are completely continent during the day. At the age of 8 years, the patient with Trisomy 21 begins weaning off the diaper. In addition, two children are too young to be weaned from diapers (Table 3).

### 3.5. Vitamin, Iron, & Electrolyte Status

Seven individuals had their vitamin and electrolyte levels checked regularly. While vitamin D replacement was required for five children, vitamin A, B12, and E replacement were required for four, three, and one child, respectively.

Urinary electrolytes were tested on a regular basis and revealed that sodium excretion was reduced in all instances. As a result, sodium was administered orally to newborns. Electrolytes were supplied utilising glucose electrolytes supplements by an oral rehydration solution after infancy. Again, after stoma closure, the need for sodium substitution decreased. Only one patient out of seven has normal sodium and potassium excretion values. Four patients continue to receive oral rehydration solution. In four children, iron substitution was required.

### 3.6. Perioperative Mortality and Morbidity

While perioperative mortality was 0%, perioperative morbidity was similarly low, with an average hospital stay of 11 days following reconstruction surgery. All perioperative issues were stoma-related, with four patients experiencing stoma prolapse, one experiencing parastomal hernia, and one experiencing mild stoma retraction.

Two significant issues emerged following stoma closure. They resulted in the most common reason for hospital readmission after surgery: (1) Sphincter achalasia caused defecation problems in six children, as evidenced by recurring indications of bowel obstruction or enteritis; (2) fluid loss and electrolyte imbalances, particularly during bouts of enteritis. Both issues, however, were strongly related to age, with the highest hospitalisation rate observed in the second year of life. Nine of the eleven children were affected. On the other hand, conservative treatment with intravenous fluids and antibiotics was sufficient to treat all children.

Another common complaint was increased stool frequency, which caused sleep issues and significant perianal skin maceration in seven children immediately after stoma closure.

In addition, three occurrences of pouch-related issues were noted (PRP). In one child, PRP relapsed within a year, while the other child developed transient PRP. Both patients, however, were successfully treated with antibiotic treatment, as an immunological explanation for PRP was considered unlikely. There was no histological evidence of pouchitis in any of the individuals. Complications did not necessitate revisions. There were no reports of anastomotic leakage, stricture, or fistula. One postoperative ileus was handled conservatively. Three months after reconstruction, a small submucosal defect was discovered during rectoscopy in one individual (Table 4).

## 4. Discussion

This is the largest single-center dataset of TCA patients describing the outcomes of J-Pouch repair. We operated on 12 TCA patients and closely monitored their progress. With the exception of two children, one with trisomy 21 and one girl just four weeks after total colectomy and J-Pouch reconstruction, it reveals that all children were completely weaned from diapers before their third birthday and had a reasonable continence rate overall. The average follow-up period was 51 months, ruling out any conclusions about long-term outcomes. However, a multicenter study of patients repaired using different procedures found similar results, with continence rates of 86 percent in the pouch group compared to 48 percent in the ileoanal anastomosis group 12 months after surgery [12]. Aside from stoma-related issues, which were all manageable, there were no operative complications, and no bowel obstruction or pouch dysfunction necessitated reoperation (Table 4). Early postoperative complications and enteritis-like symptoms occurred in 9 of 11 patients; the symptoms were minor and were treated with antibiotics and fluid resuscitation. As previously stated, initial enteritis or Hirschsprung-associated enterocolitis (HAEC) is a relatively unavoidable complication in HD patients because these patients are prone to recurring episodes of enterocolitis after primary HD repair [13]. The risk of enterocolitis increases with the length of the aganglionic segment and is highest in those with TCA [14]. Furthermore, HAEC is associated with sphincter achalasia (6/11), which can be treated with bowel control or in conjunction with Botox injection. HAEC episodes, in general, will fade over time [11]. Perianal dermatitis, which responds well to zink ointments and improves over time as stool frequency reduces, is another concern in the early postoperative period following total colectomy, independent of anastomosis type.

When ileoanal anastomosis was compared to J-pouch reconstruction, regardless of diagnosis, it was discovered that the J-pouch construction had a higher rate of pouchitis in the first few years after the reconstruction, but continence scores were significantly improved 24 months later when compared to straight ileoanal anastomosis [12]. However, most of the patients in this study had undergone surgery for UC, which has a high rate of pouchitis due to the underlying disease. Pouches in UC are responsible for 10% of pouch failure [13]. Pouchitis is an idiopathic inflammatory illness that can occur up to 50% of the time following total colectomy and J-pouch reconstruction for UC [14]. On the other hand, acute pouchitis is uncommon in primary non-inflammatory disorders such as FAP or HD [15]. This study suggests that pouchitis is unlikely following complete colectomy for TCA. Further this study found that J-Pouch reconstruction after restorative proctocolectomy for TCA in newborns is safe, with a minimal risk of early postoperative complications with very low pouchitis rates [12]. As a result, in the case of non-CU disorders, we advocate referring to pouch-related problems (PRP) rather than pouchitis. We feel that with operating experience and the avoidance of technical, operational errors such as long cuff, long blind loop, prolonged pelvic dissection, and avoiding anastomotic strictures due to compromised blood flow, these experienced difficulties can be minimised. In adolescence, the pouch size should be no larger than 14 cm; hence, while constructing a J-pouch in young children, the growth of the gut should be kept in mind, and the pouch should be generally smaller. Our cohort’s average J-Pouch size was seven centimetres (5–10 cm). We only had one patient with intestinal obstruction after J-pouch construction. This is lower than has been reported for other cohorts [13]. The main point is that massive pelvic dissection increases the possibility of intestinal bowel obstruction [15]. This can be reduced, however, by using minimally invasive approaches.

Increased frequency of bowel movements is a serious and possibly debilitating condition after J-Pouch repair [16]. Both toddlers and adults have 5–6 bowel movements per day and 1–2 per night after surgery [17]. In TCA individuals, the frequency of daily stooling normally decreases from 5–6 in infancy to 3–4 in adolescence [18]. In our study, a median 24-h bowel movement frequency of 4.5 was reported after 51 months of follow-up, which is an excellent result. Furthermore, none of them had regular nighttime bowel motions. Patients should have at least 4–5 bowel movements each day, since lower rates increase the risk of enteritis-like symptoms due to bacterial overgrowth. As a result, in these cases, patients must be encouraged to visit the toilet so that their bowels can be evacuated.

Aside from the immediate postoperative outcomes, the medium and long-term out-comes of pouch repair appear to favor J-Pouch reconstruction, which is consistent with the development in adult visceral medicine [12]. However, reconstruction after total colectomy in children with TCA is still being discussed in the literature [10]. There are numerous procedures described, but only a few centres use J-Pouch reconstruction as in adult medicine as a primary approach [19].

This is unfortunate, because pouch reconstruction provides the highest quality of life, with an average of 5–6 bowel movements per day and complete continence in more than 90% of adults (n = 553,554). After total colectomy for TCA, the J-Pouch should be the recommended surgical technique. The primary reason for this is that it is simple to build and, in the long run, produces functional results equivalent to other procedures [20].

A problem that has yet to be overcome is determining whether a colectomy and J-pouch reconstruction are appropriate for the patient and when an ileostomy closure is the best option. We believe that all patients receiving TCA should have a prophylactic ileostomy to reduce postoperative complications such as anastomotic leakage. However, our findings show that early ileostomy closure is associated with more hospitalizations, resulting in insufficient emptying and an increased risk of bacterial over-growth of the small intestine and PRP [20]. This effect appears to be independent of the reconstruction technique, as it has already been described by other researchers following TCA correction [12]. As a result, we keep the ileostomy at least three months.

## 5. Conclusions

Due to impaired function, a colectomy with a direct ileoanal repair is not an option in adult visceral surgery. Our findings and the current literature support J-pouch reconstruction in TCA patients. However, more research will be needed to determine the best time to undergo pouch surgery and ileostomy closure in TCA patients.

## 6. Limitations

The current study may be limited in accuracy due to missing data in the charts due to the study methodology. Furthermore, the small sample size and the study’s single center retrospective nature limits the conclusions derived from the current study.

## Figures and Tables

**Table 1:** Patient demographics (total n = 12).

Characteristic	n (%)	Mean (Range)
male (n)	9 (75.0)	
age at diagnosis (d)		339 (1–1872)
age at operation (d)		496 (104–1951)
age at stoma placement (d)		192 (1–1153)
age at stoma closure (d)		613 (204–2027)
follow up (m)		47 (1–100)
mortality (n)	0 (0.0)	
surgical data		
operation time (min)		270 (171–402)
pouch length (cm)		7 (5–10)
length of hospital stay (d)		11 (8–19)

d = days, m = months, min = minutes, cm = centimeter. Total ranges given for median values. Length of hospital stay and pouch length only available for n = 11 patients. No stoma needed in n = 1 patient.

**Table 2:** Patient symptoms and functional outcomes.

Nr	Sex	Age at Diagnosis (d)	Presenting Symptoms	Comorbidites	Age at Operation (d)	Height at Operation (cm)	Weight at Operation (kg)	Pouch Length (cm)	Age at Last Follow Up (y)	Stool Frequency (Per Day)	Fecal Continence	Urinary Continence	Nutrition Status
1	m	42	distended abdomen, bilious vomiting	-	178	65 (19)	7, 1 (26)	5	3	2–3	yes	yes	good
2	m	3	delayed meconium passage, distended abdomen, bilious vomiting	-	265	68 (4)	7, 1 (4)	6	5	1–2	yes	yes	good
3	m	1872	constipation, failure to thrive	-	1951	105 (3)	17, 6 (17)	7	10	1	incomplete	incomplete	good
4	m	9	distended abdomen, bilious vomiting, failure to thrive	-	115	60 (17)	5, 2 (7)		7	3–5	incomplete	yes	good
5	f	1	delayed meconium passage, distended abdomen, bilious vomiting	-	350	74 (33)	7, 9 (3)	6	8	6–8	yes	yes	good
6	m	1119	distended abdomen, constipation, failure to thrive	-	1153	90 (3)	12, 1 (6)	7	6	4–8	incomplete	yes	good
7	m	5	delayed meconium passage, distended abdomen, bilious vomiting	Trisomy 21	133	61 (13)	6 (17)	5	7		no	no	good
8	m	7	delayed meconium passage, distended abdomen, bilious vomiting	Hermelin-Mayer-Wunderlich	304	69 (4)	8, 4 (19)	7	4	6	yes	yes	good
9	m	277	delayed meconium passage, distended abdomen, bilious vomiting, constipation, failure to thrive	Renal agenesia	104	62 (56)	5, 6 (24)	10	7	6	yes	yes	good
10	m	312	delayed meconium passage, distended abdomen, bilious vomiting, constipation, failure to thrive	-	321	75 (52)	7, 6 (4)	7	1		no	no	good
11	f	167	delayed meconium passage, distended abdomen	-	581	83 (35)	10, 7 (24)	7	2	10	no	no	good
12	f	256	delayed meconium passage, distended abdomen, bilious vomiting, constipation, failure to thrive	Congenital myopathy, dysplastic kidney	498	76 (3)	7, 5 (<1)	7	1		no	no	good

d = days, y = years, kg = kilogram, cm = centimetre, m = male, f = female. Percentile for hight and weight at operation given in braces.

**Table 3:** Postoperative Outcome (total n = 12).

Outcome Parameters	n (%)	Median (Range)
stool frequency per day		4 (1–10)
fecal continence		
total continent	5 (42.7)	
partly continent	3 (25.0)	
not toilet trained	4 (33.3)	
urinary continence		
total continent	7 (58.3)	
partly continent	3 (25.0)	
not toilet trained	2 (16.7)	
good nutrition status	12 (100.0)	
normal electrolyte status (blood)	7/8	
normal electrolyte status (urine)	1/8	
high potassium	5	
low sodium	5	
normal iron status	6/6	
normal vitamin status	4/7	
need for substitution	7/9	
electrolyte	5	
vitamine A	4	
vitamine B12	3	
vitamine D	5	
vitamine E	1	
zinc	2	

Stool frequency is given as median with total range and was only available on n = 9 patients. Data for electrolyte, iron and vitamine status at last follow up was not available for all patients at the time of last follow up. Number of patients with potassium or sodium changes is given for all patients with urine electrolyte dysbalance (n = 6). Number of supplements is given for all patients requiring a substitution (n = 6).

**Table 4:** Number of peri- and postoperative complications (total n = 12).

Type of Complication	n (%)
stoma related	
prolapse	4 (36.4)
parastomal hernia	1 (9.1)
stoma retraction	4 (36.4)
pouch-related problems	3 (27.3)
enteritis	9 (81.1)
sphincter achalasia	6 (54.5)
perianal excoriations	7 (63.6)
bowel obstruction	1 (9.1)
surgical revision	0 (0.0)

Stoma-related problems were included from stoma placement to stoma closure. Non-stoma related complications were included from definitive surgery to end of follow up. One patient with only one month of follow up was not included. This patient had none of the named complications.

## Data Availability

The data described in this study are accessible from the corresponding author upon request. Due to ethical and privacy constraints, the data are not publicly available.

## Abbreviations

FAP	Familial adenomatous polyposis
HAEC	Hirschsprung-associated enterocolitis
HD	Hirschsprung Disease
PRP	Pouch-related problems
TCA	Total colonic aganglionosis
UC	Ulcerative colitis
